# Thomas Bewley, CBE, MD, FRCPI, FRCPsych (Hon)

**DOI:** 10.1192/bjb.2022.80

**Published:** 2023-06

**Authors:** Martin Mitcheson

Formerly Consultant Psychiatrist, Tooting Bec, St George's and St Thomas’ Hospitals, London, UK


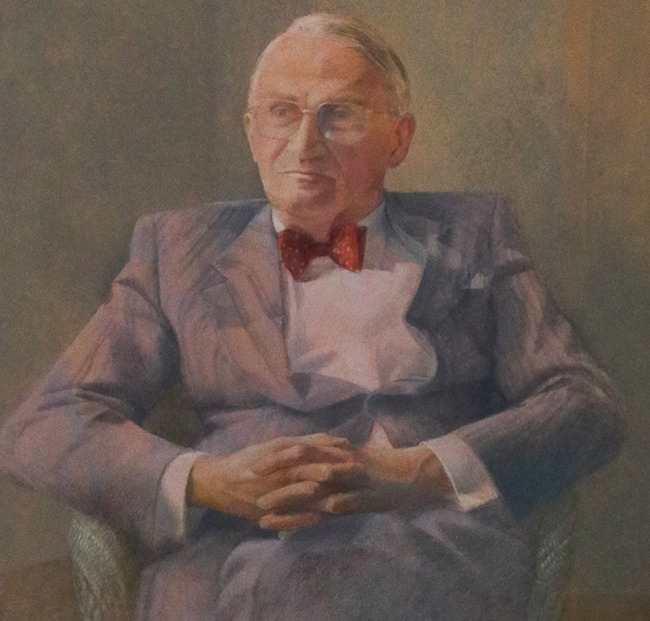


Shortly after his appointment to a consultant psychiatrist post at Tooting Bec Hospital in 1961, Thomas Bewley, who died recently at the age of 95, began to accept patients with alcohol problems as well as small numbers of heroin addicts. From this latter experience, in 1964 he published a seminal paper in *The Lancet*, drawing attention for the first time to a group of young drug users.^1^ He reported that they differed from the previously described addicts in middle age, who had become addicted to analgesics in the course of pain control or were healthcare professionals with easy access to pharmaceuticals. He recognised that the somewhat laissez-faire prescribing of heroin and cocaine by certain doctors in private practice and a few general practitioners was contributing to the increasing number of younger addicts. His treatment approach recognised that immediate abstinence was rarely easily achievable and that the management of addicts’ physical and social problems was essential – a policy now referred to as harm reduction. His experience and research in this area of addiction management, and his evidence to the Interdepartmental Committee on Drug Addiction (the Brain Committee), were to be hugely influential in the provisions of the Misuse of Drugs Act 1971, which led to the restriction of prescribing controlled drugs for the maintenance of addiction to specially licensed doctors, and to the establishment of specialist drug dependency units. He himself established such clinics at both St Thomas’ and St George's Hospitals, London, in addition to the in-patient unit he ran at Tooting Bec Hospital.

Alongside his work in addiction, Thomas became involved in the newly established Royal College of Psychiatrists and, first as Sub-Dean and then as Dean, he worked to extend and improve the training of psychiatrists working in services that were not attached to established teaching hospitals. He also furthered the promotion of the addiction specialist group to the status of a College Faculty. In 1984 he was elected President of the Royal College of Psychiatrists, the first President not to be an academic. During his presidency, he established the College Research Unit to further the development of evidence-based treatments. He was also appointed an adviser on drug dependence to the World Health Organization. In 1988 he was awarded the CBE.

Thomas Bewley was born on 8 July 1926 into a medical family, the son of Geoffrey Bewley and Victoria Bewley (née Wilson). His grandfather and father were prominent Dublin doctors and sometime superintendents of the Quaker Mental Hospital there. His mother had trained in medicine and his sister followed the family tradition and became a psychiatrist. Thomas trained in medicine at Trinity College, Dublin, and in psychiatry at St Patrick's Hospital. A separate branch of the Bewley family were tea and coffee importers who had been the first to challenge the monopoly of the East India Company, and to this day they claim that their Quaker values determine their business practice.

Thomas married Beulah (née Knox), later Dame Beulah, a distinguished epidemiologist, in 1955, having come to work in England in 1954. They had five children. He worked first in Claybury Hospital and then, after three unsuccessful applications, was accepted in 1956 at the Maudsley Hospital, London. Here he learned the need for precise formulation of a patient's management under the exacting supervision of Felix Post and developed an interest in alcohol addiction under D.L. Davis.

In 1957 the Bewleys moved to the USA, where Thomas studied problems of alcoholism in different ethnic groups at the University of Cincinnati. On returning to the UK, he undertook locum posts as a senior house medical officer at Tooting Bec Hospital and in 1961 was appointed a consultant psychiatrist there.

Thomas Bewley developed an approach to addiction that viewed the addict as a patient in need of all round medical care and treatment. He described himself as a ‘Quaker atheist’ and his approach to clinical practice was based on Quaker values of tolerance and care in the tradition of the Retreat at York. He was both a dedicated clinician and one who worked tirelessly to establish his approach to alcoholism and drug dependency across the whole country and to improve and maintain standards in psychiatry. He will be remembered also as a sympathetic and encouraging mentor of trainee psychiatrists and, perhaps more importantly, by a diminishing number of younger colleagues, for his gentle and tactful advice as they embarked on consultant responsibilities.

Somehow, alongside this busy and distinguished professional career, Thomas (never ‘Tom’!) maintained a vigorous and eclectic intellectual life. He was an avid reader, a possessor of an extensive library, a lifelong theatre and opera goer, a bridge player and a formidable chess competitor. He was the author of witty contributions to the College *Bulletin* as ‘Ezra the Scribe’ and wrote a history of the College entitled *Madness to Mental Illness*.^2^

Thomas died on 26 June 2022. Beulah died in 2018. Their daughter Sarah died in 2003. He is survived by his other four children, Susan, Louisa, Henry and Emma, and his granddaughter Hannah.
